# The effect of bivalve filtration on eDNA-based detection of aquatic organisms

**DOI:** 10.1371/journal.pone.0222830

**Published:** 2019-11-13

**Authors:** Ryan Friebertshauser, Kurtis Shollenberger, Alexis Janosik, Jeffrey T. Garner, Carol Johnston

**Affiliations:** 1 Department of Fisheries, Aquaculture, and Aquatic Sciences, Auburn University, Auburn, AL, United States of America; 2 Department of Biology, University of West Florida, Pensacola, FL, United States of America; 3 Division of Wildlife and Freshwater Fisheries, Alabama Department of Conservation and Natural Resources, Florence, AL, United States of America; University of Hyogo, JAPAN

## Abstract

As the use of environmental-DNA (eDNA) expands as a method to detect the presence and quantity of aquatic taxa, factors potentially impacting the efficacy of this technique must be investigated. Many studies have examined the effects of abiotic parameters on the degradation of environmental-DNA (e.g. UV radiation, pH, temperature, etc.), however, few have focused on biotic effectors. Through high-filtering rates coupled with dense colonization, Asian clams (*Corbicula fluminea*) are able to drastically alter the quantity of particulate matter through translocation into the sediment, potentially including sources of eDNA in lotic and lentic systems. Using a longitudinal, laboratory experiment, we tested the effect of varying densities of Asian clams on the translocation rate of common goldfish (*Carassius auratus*) DNA. Target DNA in testing tanks was quantified through quantitative PCR (qPCR) at regular intervals and compared. Tanks housing the highest density of Asian clams produced significantly lower DNA concentrations over time compared to tanks of lower densities. These results show, for the first time, a density-dependent reduction of local eDNA sources by bivalve filtration that may lead to the obstructed detection of target species through the sampling of eDNA. Based on these findings, we recommend highly concentrated bivalve populations be taken into consideration when choosing the time and locality of eDNA sampling efforts.

## Introduction

One of the first steps in conserving imperiled taxa is to gain a better understanding of their distributions and abundances [1]. The difficulty of conserving imperiled taxa, however, is contingent upon the taxa in question, its abundance, and its respective habitat [2]. Empirically sampling for aquatic individuals with low populations in large systems can often make gathering necessary distribution and abundance data challenging and/or impractical. However, a relatively new technique to assist with these issues is the collection and use of aquatic environmental DNA (eDNA). This forensic technique, aimed at qualifying or quantifying extraorganismal genetic material (e.g. feces, slime, sloughed scales, etc.) [3], has produced relatively rapid, less invasive, and more efficient detection of rare and imperiled species compared to empirical sampling efforts [4–6]. In addition, eDNA has been used to detect the invasion of harmful species earlier than previously possible, thereby increasing the chances of eradication and control [7]. While biological sampling via the collection of eDNA has produced many conservation-oriented results, successful detection of target species depends on the persistence and availability of genetic material in any given system. In order to efficiently guide sampling efforts towards optimal locations and times, conditions affecting the detectability of eDNA must be further investigated.

To understand the persistence and detectability of DNA in aquatic systems, investigation of the factors that impact detection is necessary [8]. DNA has been shown to degrade exponentially in aquatic environments [9], however these rates are highly variable across the literature [10,11]. A variety of factors such as pH, chlorophyll, temperature, and microbial load have been linked to increased degradation rate [12–14]. When viewed collectively, these results can be used to make more accurate decisions about when and where to collect eDNA samples. While understanding these causes of increased eDNA degradation is necessary for the effective use of this molecular monitoring technique, it is possible that effects of sympatric macrobiota may also serve to negatively impact detection probabilities through enhanced degradation or translocation of genetic material.

Freshwater bivalves, such as the invasive Asian clam (*Corbicula fluminea*), are known to drastically alter the makeup of aquatic environments. Through high population densities and community predominance [15], Asian clams primarily alter their local environment by transferring large quantities of matter from the water column to the sediment [16]. The size of particulate matter filtered out of the water column can range between 5,000–30,000 μm^2^ which partially includes the range of known sizes of eDNA seston [17,18]. For these reasons, it is possible that extraorganismal genetic material may be consumed or rapidly translocated into the sediment via filtration by high densities of Asian clams. A recent study investigated the effects of zebra mussel (*Dreissena polymorpha*) filtration on eDNA-based detection of target macroinvertebrates and found no significant effect [19]. However, this work used bivalve densities well below typical peak population size estimates (46 individuals/m^2^) and measured proportion of positive detections instead of quantifying DNA concentrations. Therefore, the aim of this project was to further investigate this phenomenon with finer resolution using environmentally relevant densities.

Through a longitudinal laboratory experiment, this study examined the effect of varying densities of Asian clam on the removal of goldfish (*Carassius auratus*) DNA from the water column. DNA concentrations measured via qPCR were collected over time and across tanks with varying densities of Asian clams. We hypothesized that tanks housing the highest densities of Asian clam would produce the highest rates of DNA translocation leading to reduced eDNA detections. Data were collected in order to better understand potential constraints on accurate and repeatable sampling of eDNA, especially in light of macroorganism influence on eDNA persistence.

## Materials and methods

### Study design

Asian clams were collected from Chewacla Creek in Chewacla State Park in Lee County, Alabama (32.552364°N, -85.475315°W) via a metal sieve (1x1 cm mesh) and housed in eight, 190-liter tanks, each containing 148 liters of dechlorinated tap water. Permission to collect clams was granted by employees of Chewacla State Park. Individuals were measured at the deepest point dorsoventrally and clams that fell out of a pre-determined size range (6.77–15.74 mm) were excluded from the experiment. Tanks contained two air stones to promote homogenization of DNA and in order to optimize DNA longevity, also included water heaters to control temperatures (~22° C), lacked filtration, and maintained a pH of 8 [20]. Tanks consisted of four different treatment densities of Asian clams, each with one replicate. Densities included 0, 102.56, 307.69, and 679.49 individuals/m^2^ (i.e. “Control”, “Low”, “Medium”, and “High”). Clams were given a 14-day acclimation period in which they were fed Shellfish Diet 1800® (Reed Mariculture, Campbell, CA) daily. To control for exogenous microbial loads brought in by clams, control tanks initially housed 50 individuals which were then removed upon the beginning of the experiment. Tanks contained filters during acclimation to maintain water quality. Individuals were not fed during the observation period to promote consistent filtration. All tanks experienced a 12:12 photoperiod.

After the 14-day acclimation period, tanks were spiked with three liters of common goldfish (*Carassius auratus*) DNA-enriched water that was generated by holding 30 goldfish in 60 liters of dechlorinated tap water for ten days. Due to the variable rates of eDNA shedding among individuals [21,22], we decided spiking tanks with enriched water would produce the most consistent distribution of genetic material across testing tanks. An alternate technique would be to include live fish in the testing tanks for the duration of the experiment, however, the ostensibly constant shedding rate of live animals would have not allowed us to observe natural decreases in eDNA detection in control tanks. Goldfish were sourced from a local pet store and fed flake food (TetraMin Plus Tropical Flakes ®, Tetra, Spectrum Brands, Inc.) once per day. Goldfish housing tanks were not filtered in order to limit the potential removal of genetic material. Immediately following the 10-day enrichment period, individuals were euthanized with an overdose of tricaine methansulfonate (ms222).

Water sampling began immediately after tanks were spiked. Seventeen eDNA water samples per test tank were collected over eight days. Four water samples were colledcted per day for the first three days followed by one sample a day for the remainder of the experiment (Table 1). This sampling regimen was designed to capture finer resolution during the first three days when most rapid degradation is thought to occur [13,14,18,19]. The eDNA collection protocol used was similar to that of Ficetola et al. [23] and Thomsen et al. [9] where three, 15 mL water samples were collected from the surface of tanks at each time step. Individual samples were composed of triplicate collections in order to better represent the state of the tank at each time step. In order to preserve genetic material, 1.5 mL of sodium acetate 3M buffer and 33 mL of 95% ethanol were added to samples immediately after collection. At each time step, a control sample consisting of the 3M buffer, ethanol, and 15 ml of deionized water was collected in the study room in order to test for cross contamination during the collection period. Water samples were stored at room temperate until extraction. Clams were euthanized via freezing at the termination of the experiment. This study, including Goldfish euthenasia, was approved by Auburn University’s Animal Care and Use Committee (protocol #2017–3079).

**Table 1:** Sampling regimen used throughout experiment.

Day	1	2	3	4	5	6	7	8
Time	0600	0600	0600	1200	1200	1200	1200	1200
Time	1200	1200	1200					
Time	1800	1800	1800					
Time	2400	2400	2400					

### DNA extraction and qPCR

DNA was extracted from preserved water samples using the DNeasy® Blood and Tissue Kit (Qiagen, Inc., Valencia, CA). In order to produce a pellet for extraction, triplicate samples were first centrifuged for 30 minutes at 4°C and 3500 RPM. The supernatant produced from centrifugation was then discarded and the remaining pellet and ethanol from sample triplicates were pooled into a 1.5 ml tube with a sterile pipette. Pooled samples were then centrifuged at 14000 RPM for 3 minutes. DNA extraction from this point forward followed the DNeasy® spin column protocol. Extraction blanks were included to ensure cross contamination did not occur during the DNA extraction process. Extracted DNA samples were stored at 4°C until qPCR analysis.

A Goldfish primer set [24] was used to assess the concentration changes of mitochondrial DNA (mtDNA). The primers (F: 5’ CCCACAACCTAAATATCGTTACC 3’; R: 5’ TCTTTCCTTTGTTGCACTCC 3’) amplified a short 51 base pair segment within the 16S rRNA of mtDNA. A relatively short primer was used as small DNA fragments are known to decay at a slower rate compared to longer fragments [25] allowing for the potential of a longer DNA detection duration. Quantitative PCR assays were performed with a QuantStudio^™^ 3 Real-Time PCR System (Applied Biosystems, Foster City, CA). Assays were performed in a 12.5 μl reaction consisting of 4.75 μl of Milli-Q® water, 0.25 μl of forward primer, 0.25 μl of reverse primer, 6.25 μl of SYBR ™ Green Master Mix (Applied Biosystems, Foster City, CA), and 1 μl of DNA template. The following PCR conditions were used: 50°C for 2 min, 95°C for 2min, followed by 40 cycles of 95°C for 15 seconds and 60.2°C for 1 min. The QuantStudio Design and Analysis program (Applied Biosystems, Foster City, CA) was used to generate raw cycle threshold (CT) values. Each sample was run in triplicate. Negative PCR controls containing only the reaction mix were included on each qPCR plate. Tissue derived goldfish DNA was also analyzed to ensure proper amplification of target DNA.

### Statistical analysis

There are currently no standards within the eDNA literature that designate the amount of eDNA assay replicates required to determine a sample as positive [11]. Samples analyzed by qPCR in a recent study were considered positive if 1/12 assay replicates were successfully amplified [26]. However, more conservative thresholds may be necessary to promote consistency in results [27], especially when attempting to quantify target DNA. Therefore, to reduce variance between qPCR replicates, a threshold of reliable detection at 2/3 qPCR replicates was decided upon. Assayed samples that fell below this threshold were not included in analysis. The raw CT values for replicates at or above this threshold were averaged and the inverse of this average was used for analysis in order to aid interpretation and is hereafter referred to as CT-units.

In order to compare the effects of variable tank densities on eDNA concentrations, a general linear model including tank density (fixed factor with three levels), time (continuous factor), temperature at each time-step (continuous factor), and a tank density by time interaction term was used. In order to produce interaction contrasts not presented in the standard table of coefficients, a general linear hypothesis test was used. Detection of significant interaction terms were used to support the hypothesis of density-dependent concentrations of DNA over time. Statistical analyses were conducted using R version 3.3.4 (R Core Development Team, 2016) and interpreted with an alpha criterion of 0.05. Results were reported as effect size +/- 95% confidence interval (CI). Normality of data was observed visually using residual-fitted plots.

## Results

**Fig 1:**
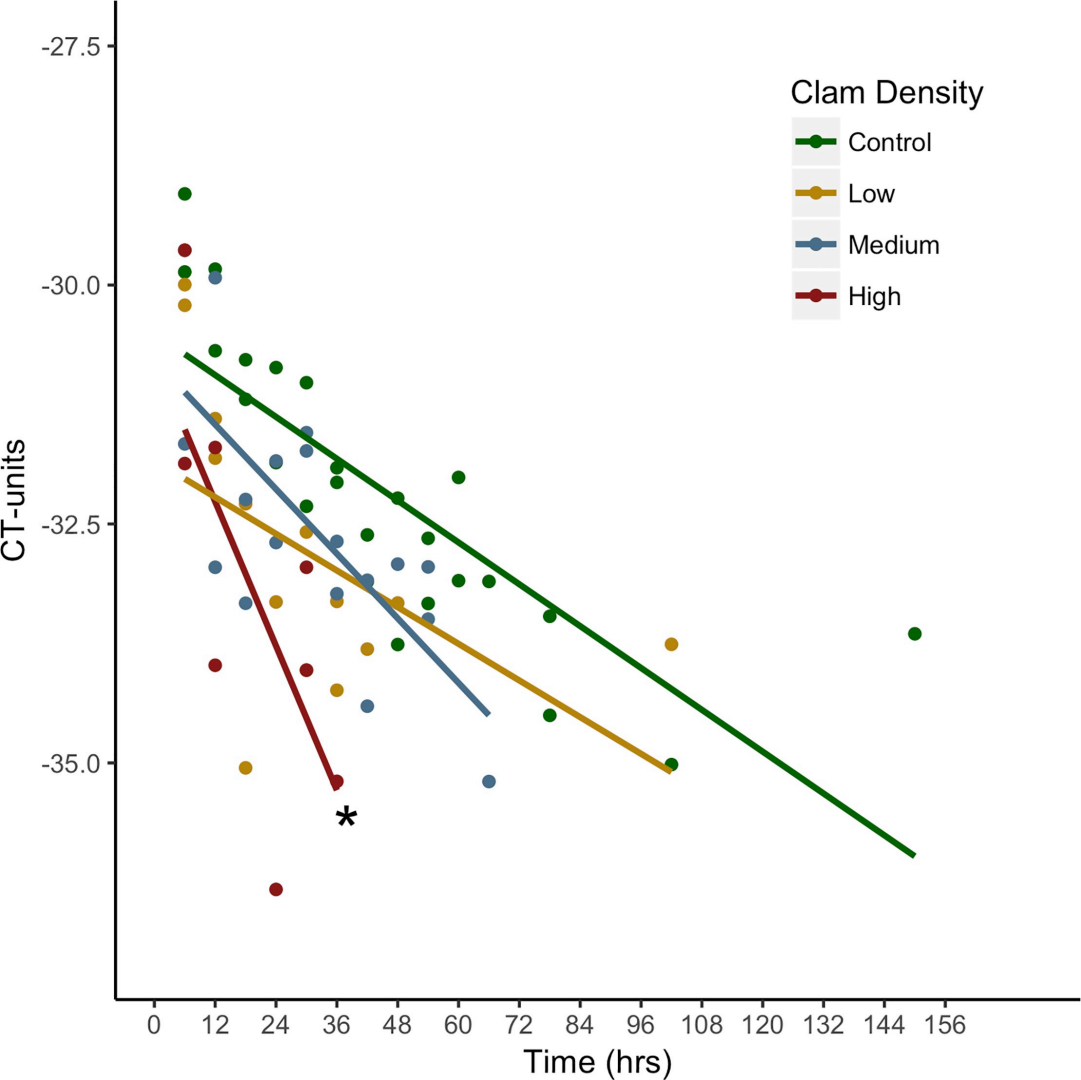
Concentration of DNA (in CT-units) across treatment densities over time. CT-units measured at each timestep across four treatment densities (Control = 0 clams/m^2^, Low = 102.56 clams/m^2^, Medium = 307.69 clams/m^2^, and High = 679.49 clams/m^2^). Asterisk (*) denotes significantly different slope compared to all other treatment densities. (n = 2).

The model testing the effect of varying clam densities on eDNA concentrations produced significant interaction terms such that high-density tanks decreased by 0.134 (+/-0.0666 CI), 0.139 (+/-0.0689 CI), and 0.11007 (+/-0.07101 CI) more CT-units per hour than control, low and medium density tanks respectively (p = 0.000152, 0.000142, & 0.0029). These interaction terms represent significantly different slopes across densities and therefore significantly different rates of DNA removal from the water column (Fig 1). Interaction terms were not statistically significant for low and medium densities compared to control densities (p = 0.743 & 0.1608).

As experiments were not carried out in a climate-controlled environment, tank temperatures ranged between 18.9 and 24.8°C over the duration of the experiment. Due to these fluctuations, we observed an average effect of temperature such that CT-units decreased by 0.73 (+/-0.477 CI) units with each one-degree increase in temperature. During qPCR assays, we observed samples from all tanks falling below the detection threshold (2/3 amplifications) then proceeding to amplify at or above the threshold in subsequent time steps, hereafter referred to as intra-trial gaps. Therefore, to summarize duration of detection and final translocation of DNA away from the water column, the last timestep to amplify was determined at or above the set threshold as an endpoint. Once endpoints were reached, control, low, medium and high-density tanks had decreased by 16.091, 18.870, 22.410, and 29.340% CT-units, respectively, compared to time zero. The durations of detection for control, low, medium, and high-density tanks were 150, 102, 66, and 36 hours, respectively, into the experiment. No collection or extraction controls were successfully amplified by qPCR, thereby indicating that no cross contamination of target DNA occurred in the field or laboratory setting.

## Discussion

This study examined the effect of varying densities of Asian clams on the removal of target DNA from the water column. The significantly higher translocation rate observed in high-density tanks compared to control tanks support the hypothesis of bivalve-induced decay of extraorganismal genetic material and refutes the findings of a recent study investigating a similar hypothesis with relatively low densities of Zebra Mussels [19]. Likely the critical difference between this recent Zebra Mussel study and the data presented here is the densities of bivalves used in treatments, especially considering that Zebra mussels have been observed exhibiting greater filtration rates compared to Asian clams [28].

Regardless of intra-trial gaps, our data support this density-dependent effect. These gaps in data, when viewed independently, would be considered a non-detection, but when observed over a temporal scale appear to simply be false negatives. This lack of precision, inherent to eDNA work, may arise through the subsampling of heterogeneous environments in the field or lab. Whether a sample is taken from a large body of water or an extracted sample, an uneven distribution of target DNA, especially in low concentrations, can produce non-detections based on chance alone [29]. These results clearly support a clam density-dependent removal of eDNA from the water column. However, it cannot be determined if this relationship is caused by degradation or translocation of pelagic genetic materials to the benthos or other locations.

Considering that Asian clams are known to actively deposit both rejected and ingested (via excretion) seston, which approximate the size of eDNA substrates [17,18], into the sediment [30–32], this study demonstrates that filtration is most likely the mechanism responsible for the increased rate of eDNA removal observed. Additionally, water quality parameters known to alter DNA decay rate, such as temperature and pH, were either kept constant across tanks or accounted for in the statistical model used for this study. An alternative explanation is that the ostensibly higher microbial load of high-density test tanks, could have partially accounted for increased decay rate through DNA transformation or degradation by bacteria [33,34]. Future studies should seek to further investigate this pelagic/benthic coupling through the collection of both water and sediment eDNA samples while controlling for microbial abundances.

The fact that only high-density tanks significantly decreased eDNA concentrations over time suggests a density-dependent threshold for this phenomenon. While this pattern is seen in laboratory settings, understanding how these results translate to natural settings may prove difficult. Obvious factors such as increased water volume and flow will make these relationships less clear. Additionally, the densities of clams used in this experiment were large enough to elicit a response, but are still far below the 3,000–16,000 individuals/m^2^ previously reported in some natural aquatic systems [35,36]. The filtration rates of bivalves are also known to be highly variable as well as seasonal, with highest filtration in warmer months [37]. Relationships between these variables and natural settings should be further investigated in order to better understand this potential hindrance to sampling.

These results present, for the first time, local removal of pelagic extraorganismal DNA by sympatric species and have strong implications for the sampling of eDNA in these settings. The removal of pelagic DNA by bivalve filtration may reduce eDNA concentrations such that negative-detections are generated when the target is actually present; leaving target individuals virtually undetectable. Specifically, the density-dependent effects observed in this study may impart greatest impacts on migratory or low-abundance taxa where eDNA concentrations are low due to the numbers or time of residence. With further investigation into the factors affecting bivalve-induced removal of genetic material (i.e. filtration rates, densities, etc.), this information will assist researchers in optimizing spatial and temporal decisions regarding eDNA sampling, thereby increasing the efficacy and efficiency of this technique.

## Supporting information

S1 Master Data Plos One This .csv file includes all primary data collected and used for analysis. (CSV)
